# Simultaneous occurrence of invasive pulmonary aspergillosis and diffuse large B-cell lymphoma: case report and literature review

**DOI:** 10.1186/s12885-019-6471-x

**Published:** 2020-01-06

**Authors:** Lianyou Shao, Longxiang Jiang, Siyao Wu, Lihua Yu, Liangxing Wang, Xiaoying Huang

**Affiliations:** 1 Division of Pulmonary Medicine, Key Laboratory of Heart and Lung, The First Affiliated Hospital of Wenzhou Medical University, Wenzhou, Zhejiang, 325000 China; 2 Division of Pulmonary Medicine, Integrated Chinese and Western Medicine Hospital of Wenzhou, Wenzhou, Zhejiang, 325000 China

**Keywords:** Lymphoma, Opportunistic infection, Concurrent infection, Biopsy

## Abstract

**Background:**

Patients with lymphoma are at risk for developing pulmonary opportunistic infections due to immunocompromise. However, clinical reports of concurrent lymphoma and opportunistic infection at presentation are rare and often confined to single cases. A delayed diagnosis of either opportunistic infection or lymphoma usually occurs in this complex situation. Here, we report such a case and analyse 18 similar cases searched in the PubMed database to deepen clinicians’ understanding.

**Case presentation:**

A 48-year-old man presented with a 3-month history of fever, cough and emaciation. High-resolution computed tomography revealed bilateral cavitating lesions of different sizes. *Aspergillus fumigatus* complex was identified from a bronchoalveolar lavage fluid culture. However, antifungal treatment combined with multiple rounds of antibacterial therapy was unsuccessful, and the patient’s lung lesions continued to deteriorate. Multiple puncture biopsies finally confirmed the coexistence of diffuse large B-cell lymphoma. Despite the initiation of combination chemotherapy, the patient died of progressive respiratory failure.

**Conclusions:**

Synchronous pulmonary lymphoma and simultaneous opportunistic infection is rare and usually lacks specific clinical and imaging manifestations. Lymphoma should be considered as part of the differential diagnosis of patients with an opportunistic infection when treatment fails or other symptoms are present that could be considered “atypical” for the condition. Tissue biopsy is the gold standard, and multiple biopsies are essential for making the final diagnosis and should be performed upon early suspicion.

## Background

Lymphomas are a diverse group of clonal neoplasms arising from B and T lymphocytes and natural killer cells. Manipulation and silencing of the host’s immune system by methods ranging from changes in the cellular microenvironment composition to changes in distinct signalling pathways is an important feature of various lymphomas [1, 2]. Chemotherapeutic treatment of these diseases can also cause prolonged and profound neutropenia and immunosuppression. Therefore, individuals with lymphoma are at high risk for developing opportunistic infections. The incidence of tuberculosis (TB) has been reported to range from 0.9 to 13.2%, and that of miliary TB has been reported to be 35 times higher than in the general population [3, 4]. A retrospective study revealed that the incidence of invasive fungal infection (IFI) was 1.1% in Hodgkin’s lymphoma (HL) patients and 0.3% in non-Hodgkin’s lymphoma (NHL) patients [5]. *Aspergillus* was the most frequent causative pathogen, and most cases appeared during intensive therapy for tumours using chemotherapy, immunosuppressive agents or haematopoietic stem cell transplantation [5, 6]. However, a rare clinical scenario remains in which lymphoma and opportunistic infection exist simultaneously at presentation. The exact incidence is unknown, and it is often confined to single cases. The presence of one disease may eclipse another and thereby provide a challenge to clinicians. Here, we present the case of a patient with synchronous pulmonary aspergillosis and diffuse large B-cell lymphoma at presentation to improve our understanding of this condition.

### Case presentation

A 48-year-old male was referred to the clinic for recurrent fever, cough and 5-kg weight loss during past 3 months. He denied breathing difficulties, chest pain, night sweats, wheezing and other uncomfortable symptoms. He was a smoker of 30 pack-years and had no history of travel or TB. There were no significant findings in his prior medical or family history.

At the time of the initial evaluation, the patient showed an auricular temperature of 39.2 °C, a blood pressure of 108/70 mmHg, a pulse of 108, and a respiratory rate of 20. He had diminished breath sounds on both sides, and no enlarged lymph nodes were noted. Examinations of the heart, abdomen, extremities and nervous system were normal.

**Fig. 1:**
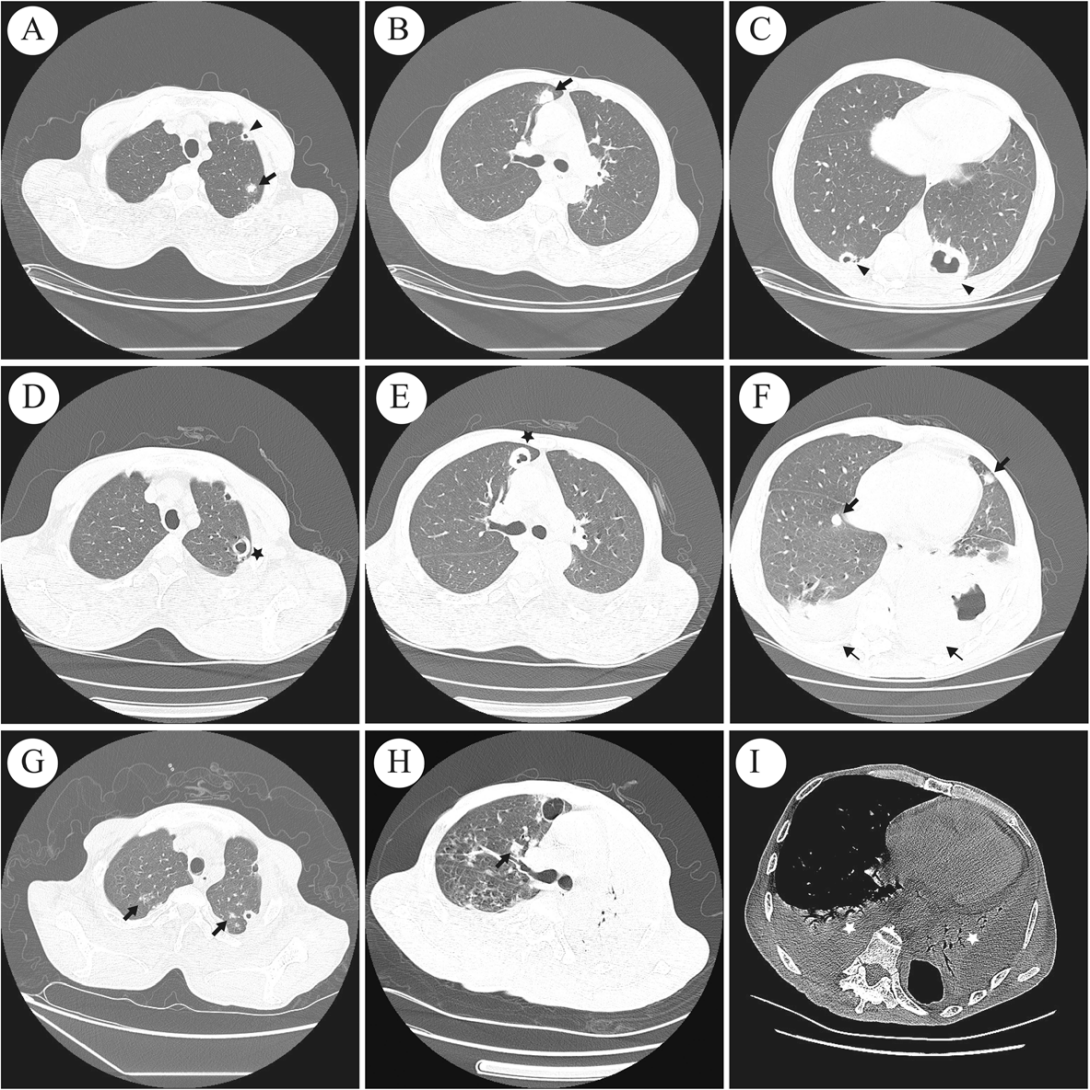
Chest CT findings during hospitalization. **a**-**c**: A CT scan of the chest (on admission) showing multiple nodules (thick arrows) and thick-walled cavities (black triangle) in lung fields as well as enlarged mediastinal lymph nodes. **d**-**f**: Subsequent chest CT (23rd day) showing new emerging round opacities (thick arrows), expending lung abscess and cavities (black stars), bilateral pleural effusion (thin arrows). **g**-**i**: Subsequent chest CT (49th day) showing increased pleural effusion, atelectasis and consolidations on both sides with air bronchograms (white star)

**Fig. 2:**
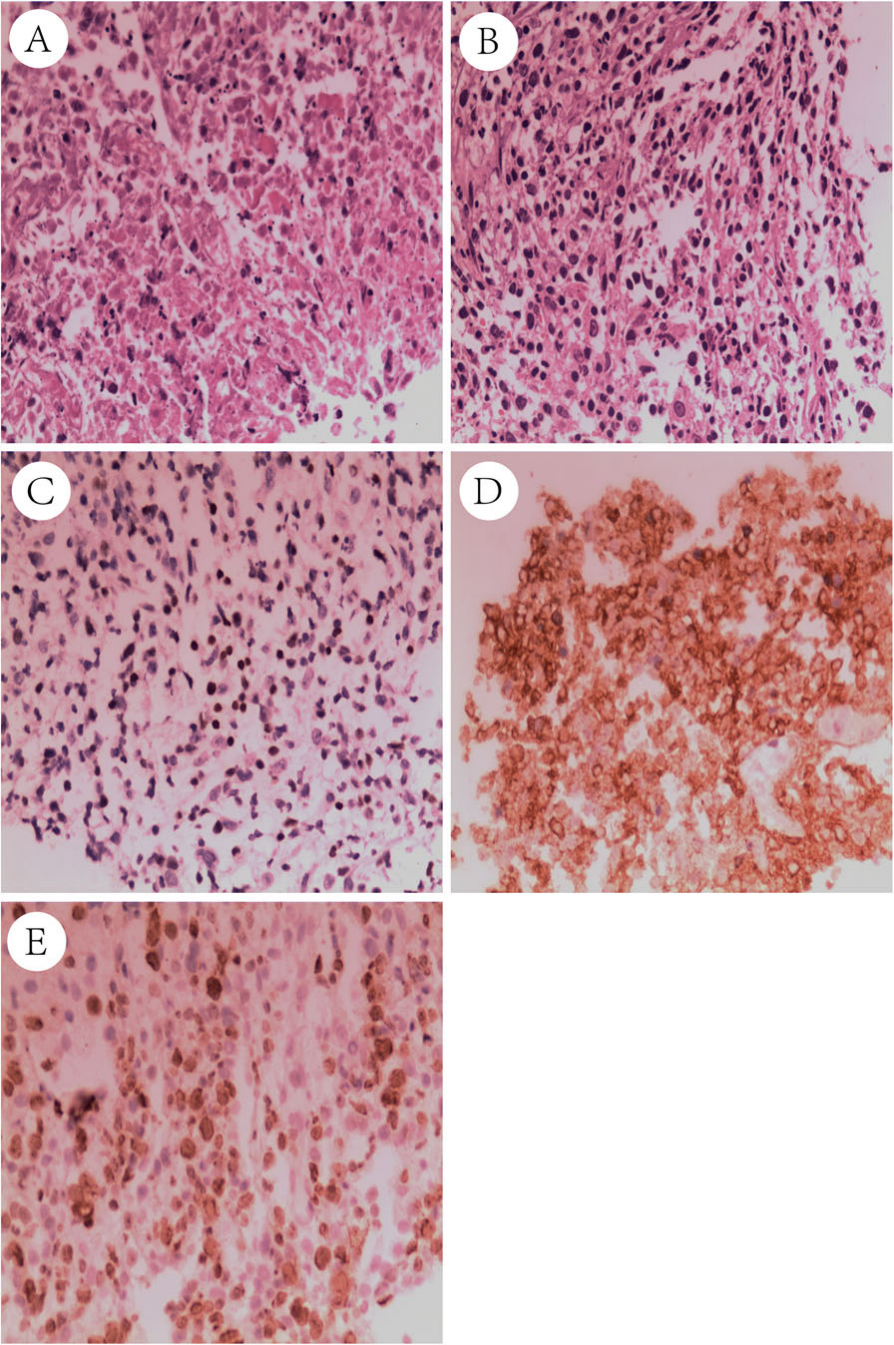
Pathological staining and immunohistochemical results. **a**-**b**: Coagulative necrosis and polygonal atypical lymphoid cell proliferation. Immunohistochemical staining shows positive markers for EBER (**c**, 400×) and CD20 (**d**, 400×) with a Ki-67 rate of 70% (**e**, 400×)

Laboratory data showed that the patient’s white blood cell count, haemoglobin level and platelet count were normal. C-reactive protein and lactate dehydrogenase (LDH) levels were elevated at 151.00 mg/L (normal range: 0~8 mg/L) and 395.00 U/L (0~247 U/L). Tumour markers, such as CEA, NSE, SCCA, ProGRP and CYFRA21-1, were all in the normal range. HIV serology was also negative. Computed tomography (CT) of the chest showed bilateral cavitating lesions with mediastinal enlarged lymph nodes (Fig. 1, A-C). The patient was started on empiric antibiotic treatment with cefoperazone-sulbactam. After 3 days of therapy, his temperature was still above 38.5 °C. A CT-guided biopsy of the pulmonary cavity was performed on the 4th day after admission. Pathology revealed mild atypical alveolar epithelioid cells and chronic interstitial fibrous tissue proliferation with necrosis. The tissue was negative on smear and culture for acid-fast bacilli. Periodic Acid-Schiff (PAS) stain was also negative. The patient’s antibiotics were changed to imipenem-cilastin sodium and metronidazole. However, the second combination of antibiotics was ineffective. The patient was still febrile, and further blood cultures remained negative. On the fifth day, he underwent bronchoscopy and bronchoalveolar lavage, which were negative for any masses, abscesses or areas of bleeding. However, the patient tested positive for galactomannan antigenemia in bronchoalveolar lavage fluid (BALF) and blood. *Aspergillus fumigatus* complex was identified from the BALF culture on the 10th day. Due to the new microbiological findings, the patient was treated with voriconazole. In addition, the patient was still treated with linezolid, moxifloxacin and imipenem-cilastin sodium combined with metronidazole successively during the third round of antibiotic use. He was persistently febrile and developed breathing difficulties. We recommended the patient undergo positron emission tomography/computed tomography (PET-CT) to assess the possibility of haematological malignancies. It was refused because of the financial burden. We performed bone marrow aspiration on the 20th day. No abnormal cells were observed in the bone marrow examination. A CT scan performed 23rd day revealed further expanding consolidation and bilateral pleural effusion (Fig. 1, D-F). Thoracentesis was performed on the 24th day. Pleural fluid analysis revealed a red blood cell count of 16,320 cells/μL and a nucleated cell count of 1280 cells/μL (31% lymphocytes and 54% segmented cells), and the Rivalta test was negative. LDH and adenosine deaminase (ADA) levels were 381.00 U/L (0~247 U/L) and 15.00 U/L, respectively. There were no malignant cells in the pleural effusion. Considering the possibility of opportunistic pathogens, additional drugs were added to cover nocardia and pneumocystic infections. However, his temperature still increased to 40 °C. A second CT-guided biopsy was performed on 27th day to find the cause of repeated fever, and the pathological examination of the specimen revealed polygonal atypical lymphoid cell proliferation with necrosis. The second PAS staining and tissue culture were also negative. Immunohistochemical staining on 35th day showed positive markers for CD20, EBER, BCL-2, PAX5, MUM-1 and a Ki-67 rate of 70% (Fig. 2), which was consistent with a diagnosis of diffuse large B-cell lymphoma (DLBCL). He also had a serum IgM test for Epstein–Barr virus (EBV) and other viruses, including influenza and toxoplasma, rubella virus, cytomegalovirus, herpes virus (TORCH), which were all negative. The EBV load was less than 5*10^3^ (< 5*10^3^). He was therefore diagnosed with synchronous pulmonary aspergillosis and DLBCL. Despite the combined R-COPE chemotherapy (rituximab 600 mg d 0 + cyclophosphamide 0.4 g d1–2 + vindesine 4 mg d1 + dexamethasone 20 mg d1–4 + VP-16 0.1 g d1–2) starting 38th days after hospitalization, the patient’s situation continued to deteriorate. He developed cervical, axillary and inguinal lymph node enlargement, which were unremarkable on admission. His LDH increased to 1627 U/L. The following CT scan (Fig. 1, G-I) showed bilateral pleural effusion, atelectasis and consolidations of all right lung lobes with air bronchograms. Then, the patient died of progressive respiratory failure on the 52nd day.

## Discussion and conclusion

Lymphoma represents a spectrum of malignant neoplasms arising from the lymphoid system with an incidence of approximately 8% of all malignancies. 25 to 40% of HL/NHL tumours arise at extranodal sites. The most common sites are the gastrointestinal tract, tonsils, skin and connective tissues. Lymphomas originating from the lung may account for 3.6% of cases [7–9]. Pulmonary lymphoma may represent primary or secondary involvement of the lungs. Primary pulmonary lymphoma (PPL) has been defined as a clonal lymphoid proliferation affecting one or both lungs (parenchyma and/or bronchi) in a patient with no clinical, pathological, or radiographic evidence of lymphoma elsewhere, either in the past or at present or for 3 months after presentation [10]. Secondary pulmonary lymphoma is more common than PPL. It refers to a secondary involvement of the lung from a known extrapulmonary lymphoma or dominant pulmonary lesion, with indolent primary extrapulmonary lesions observed within 3 months [11, 12]. Lymphoma of the lung is asymptomatic or have nonspecific respiratory symptoms, such as fever, cough, dyspnoea, chest pain, and haemoptysis. Radiological manifestations of lymphoma involving the lung are also variable. The vast majority of cases present as multiple nodules, consolidation, solitary masses or cavities with or without enlarged lymph nodes. A rare subtype of lymphoma can also present with diffuse ground-glass shadows or pleural effusion on a chest CT scan [13]. Therefore, the differential diagnosis includes TB, fungal infections, interstitial lung disease, neoplastic disease and metastatic spread from solid malignancies.

Opportunistic infection frequently develops in people with reduced immunity, such as individuals with advanced age, diabetes mellitus, HIV infection, or a history of drug abuse, organ transplantation or immunosuppresive therapy. The presentation of an opportunistic infection is related to host immunity and physiological conditions. The lung is the classical site of opportunistic infection. Disseminated infections caused by *Mycobacterium tuberculosis* and pathogenic fungi can also manifest as multifocal infiltration and lymph node masses in the image. Therefore, significant overlaps exist among opportunistic infections and lymphoma.

The patient we reported first presented with a high fever and multiple pulmonary cavities on a CT scan. Based on the findings of microbiology, the initial diagnosis was pulmonary aspergillosis, which was consistent with the imaging. However, neither antifungal therapy nor multiple rounds of antibiotic therapy were effective. The patient’s lung lesions showed rapid progression, and he developed superficial lymph node enlargement during hospitalization. A repeat biopsy of the same lesion confirmed malignant lymphoma.

The coexistence of lymphoma and opportunistic infection at the initial time of diagnosis is rare in the literature. We chose “lymphoma”, “Hodgkin’s lymphoma”, “opportunistic infection”, “fungal infection”, “tuberculosis” etc. as keywords and searched cases from 1960 to present in the PubMed database through different combinations of keywords.

Only a few publications have reported this condition (Table 1) [14–30] (see Table 1. Reported cases of synchronous opportunistic infection and lymphoma). In all 18 cases, both diseases coexisted at initial presentation, with 12 cases of concomitant TB, 2 cases of pulmonary *Aspergillus* infection, 2 cases of pulmonary cryptococcosis, 1 case of *Legionella pneumophila* pneumonia, and 1 case of pulmonary cytomegalovirus (CMV) infection (Table 2).

**Table 1:** Reported cases of synchronous opportunistic infection and lymphoma

NO.	Age ranges	Diagnosis	Author	Title	Year	Reference
1	55–60	NHL + Pulmonary cryptococcosis	Robert, K. et al.	Non-Hodgkin’s Lymphoma with Lung Lesion	1977	[14]
2	35–40	Lymphoma+Pulmonary cryptococcosis	Oka, M. et al.	A case of pulmonary cryptococcosis with diffuse pulmonary involvement of malignant lymphoma	1985	[15]
3	70–75	Lymphoma + Legionella pneumophila pneumonia	Miyara, T. et al.	Rapidly expanding lung abscess caused by Legionella pneumophila in immunocompromised patients: a report of two cases	2002	[16]
4	40–45	HL + TB	Costa, L.J. et al.	Simultaneous occurrence of Hodgkin disease and tuberculosis: report of three cases	2004	[17]
5	40–45	HL + TB	Costa, L.J. et al.	Simultaneous occurrence of Hodgkin disease and tuberculosis: report of three cases	2004	[17]
6	10–15	HL + TB	Codrich, D. et al.	Primary pulmonary Hodgkin’s disease and tuberculosis in an 11-year-old boy: case report and review of the literature	2006	[18]
7	60–65	DLBCL+TB	Sachdev, R. et al.	Coexistent Nodal Diffuse Large B-Cell Lymphoma With Extrapulmonary Tuberculosis: A Rare Case	2016	[19]
8	60–65	NHL + TB	Dres, M. et al.	Tuberculosis hiding a non-Hodgkin lymphoma “there may be more to this than meets the eye”	2012	[20]
9	65–70	T-cell lymphoma+Pseudomembranous tracheitis	Malhotra, P. et al.	Pseudomembranous tracheitis caused by Aspergillus fumigatus in the setting of high grade T-cell lymphoma	2017	[21]
10	55–60	T cell lymphoma+TB	Hashmi, H.R.T. et al.	An Unusual Triad of Hemophagocytic Syndrome, Lymphoma and Tuberculosis in a Non-HIV Patient	2017	[22]
11	25–30	HL + TB	Reddy, R. C. et al.	A case of concomitant Hodgkin’s lymphoma with tuberculosis	2014	[23]
12	15–20	HL + TB	Enteria. et al.	A Rare Case of Anterior Mediastinal and Right Lateral Neck Mass: TB With Hodgkin’s Lymphoma	2017	[24]
13	10–15	ALCL+TB	Baka, M. et al.	Successful treatment in a child with anaplastic large cell lymphoma and coexistence of pulmonary tuberculosis	2013	[25]
14	65–70	BALT lymphoma+TB	Klein, T.O. et al.	Bronchus-associated lymphoid tissue lymphoma and *Mycobacterium tuberculosis* infection: an unusual case and a review of the literature	2007	[26]
15	65–70	BALT lymphoma+Mycobacterium avium Infection	Gaur,S. et al.	Bronchus-Associated Lymphoid Tissue Lymphoma Arising in a Patient With Bronchiectasis and Chronic Mycobacterium avium Infection	2004	[27]
16	70–75	BALT lymphoma+TB	Yukinori Inadome, et al.	Malignant lymphoma of bro nchus-associated lymphoid tissue (BALT) coexistent with pulmonary tuberculosis	2001	[28]
17	80–85	NHL + pulmonary aspergillosis	Miguel G.G. et al.	Invasive pulmonary aspergillosis: a rare presentation of non-Hodgkin’s lymphoma	1994	[29]
18	65–70	NHL + CMV infection	Annunziata M. et al.	CMV infection and pneumonia in hematological malignancies	2003	[30]

*NHL* non-Hodgkin’s lymphoma, *HL* Hodgkin’s lymphoma, *TB* tuberculosis, *DLBCL* diffuse large B cell lymphoma, *ALCL* anaplastic large cell lymphoma, *BALT lymphoma* bronchus-associated lymphoid tissue lymphoma, *CMV* cytomegalovirus

**Table 2:** Analysis of information about synchronous opportunistic infection and lymphoma

Gender	
Men	12
Women	6
Age	
≤ 20	3
20–60	6
≥ 60	9
Lymphoma coexisting with	
Tuberculosis	12
Pulmonary *Aspergillus* infection	2
Pulmonary cryptococcosis	2
CMV infection	1
*Legionella pneumophila* infection	1
Clinical presentation	
Fever	9
Cough/haemoptysis	7
Superficial lymphadenopathy	5
Weight loss	4
Dyspnoea	4
Dizzy	2
Chest pain	2
Outcome	
Remission	10
Died	7
UK	1

*CMV* cytomegalovirus, *UK* unknown

This condition can happen to individuals of any age or either gender. And it seems to be more frequent in males, with a ratio of approximately 2 to 1. Only one (no. 14) of the patients had an explicit history of diabetes mellitus, and one patient had urothelial cancer (status postresection, no. 9), and the other patients had no immunocompromised statuses previously. The three most prevalent clinical presentations among such patients are fever (50.0%), cough (38.9%) and superficial lymph node enlargement (27.8%). Most chest images indicate mediastinal or hilar lymph node enlargement, solitary or multiple pulmonary nodules and cavitating lesions. Other presentations include consolidation and hydrothorax on thorax CT scans (Table 3) (see Table 3 Clinical presentations and imaging features of the chest).

**Table 3:** Clinical presentations and imaging features of the chest

No.	Age ranges	Clinical presentations								Imaging features of the chest
		Fever	Superficial lymphadenopathy	Cough/hemoptysis	Weight loss	Dyspnea	Dizzy	Chest pain	UK	
1	55–60		√							A nodule in the right lung.
2	35–40			√		√				Consolidations in both lungs and enlarged left hilar lymph node.
3	70–75	√								The first time: not remarkable.
										The second time: a nodule in the left lung.
										The third time: cavitation in consolidation.
4	40–45		√							A cavitated lesion in the right lobe.
5	40–45	√								Enlarged mediastinal lymph node.
6	10–15	√		√	√					Left lower lobe atelectasis.
7	60–65	√			√					Enlarged mediastinal lymph node and pleural effusion.
8	60–65	√		√	√		√			Enlarged mediastinal lymph nodes.
9	65–70	√			√					Enlarged mediastinal, hilar and subcarinal lymph nodes.
10	55–60	√					√			Bilateral nodules and ground-glass opacification.
11	25–30			√		√		√		Enlarged mediastinal and hilar lymph nodes.
12	15–20		√					√		Mediastinal mass.
13	10–15		√							The first time: a nodule in the left lung and enlarged mediastinal lymph node.
										The second time: two new nodules in the lung.
14	65–70								√	The first time: miliary pattern and consolidation in the lung.
										The second time: multiple masses and small cavities in the lung.
15	65–70			√						The first time: bronchiectatic change and parenchymal infiltrates in the right upper and lower lobes.
										The second time: persistent bronchiectatic change and parenchymal infiltrates with a new consolidation in the right middle lobe.
16	70–75								√	A nodular lesion with pleural thickening and several satellite lesions involving a peripheral small bronchus.
17	80–85	√		√		√				Bilateral interstitial pattern.
18	65–70	√	√	√		√				A bilateral diffuse interstitial pattern without pleural effusion.

*UK* unknown;

During the diagnostic process, the persistent clinical symptoms and new lesions are the main reasons prompting clinicians to take another cause into consideration. The diagnostic approach almost always involves various types of invasive methods, such as superficial lymph node biopsy (61.1%), bronchoscopy or BAL (38.9%), bone marrow aspiration (38.9%), transbronchial biopsy (33.3%), needle aspiration biopsy of lung lesions (27.8%), surgical operations (22.2%), mediastinoscopy (11.1%) and thoracoscopy (11.1%) (Table 4) (see Table 4 Multiple biopsy methods involved in diagnostic processes). Eighty percent of patients undergo more than one biopsy in the same or different lesions during the diagnostic process because of negative pathology results or a lack of satisfactory biopsy specimens. In only two cases (no. 7, 13), the same tissue specimen revealed *Mycobacterium tuberculosis* infection coexisting with lymphoma. Three cases (no. 2, 3,17) were diagnosed only by postmortem analysis. The time from initial onset to definite diagnosis ranged from 3 days to 14 months (Table 5).

**Table 4:** Multiple biopsy methods involved in diagnostic processes

No.	Age ranges	Biopsy methods										
		Needle aspiration or excision biopsy of lymph node	Needle aspiration biopsy of lung lesion	Bronchoscopy or BAL	TBLB or TBNA	Thoracocentesis	Bone marrow aspiration	Lumbar puncture	Mediastinoscopy	Thoracoscopy	Surgical Operation	Postmortem
1	55–60	√	√	√			√					
2	35–40				√							√
3	70–75	√		√	√		√					√
4	40–45	√								√		
5	40–45	√							√			
6	10–15			√√			√				√	
7	60–65	√				√						
8	60–65			√							√	
9	65–70	√		√	√		√√					
10	55–60	√			√		√					
11	25–30	√			√				√			
12	15–20	√	√									
13	10–15	√	√				√	√				
14	65–70		√	√								
15	65–70		√	√			√√				√	
16	70–75				√					√	√	
17	80–85											√
18	65–70	√

**Table 5:** The delayed time and prognosis of each case

No.	Age ranges	Diagnosis 1	Delayed time (days)	Diagnosis 2	Outcome
1	55–60	NHL	UK	Pulmonary cryptococcosis	Remission
2	35–40	Pulmonary cryptococcosis	139	Lymphoma	Died (respiratory failure)
3	70–75	pneumonia	29	Lymphoma + *Legionella pneumophila* pneumonia	Died (cardiac arrhythmia)
4	40–45	TB	UK	HL	Remission
5	40–45	TB	60	HL	Remission
6	10–15	TB	UK	HL	Remission
7	60–65	DLBCL	UK	TB	Remission
8	60–65	TB	14	NHL	Died (septic shock)
9	65–70	T-cell lymphoma	UK	Pseudomembranous tracheitis	Died (respiratory failure)
10	55–60	T-cell lymphoma	20	TB	Died (multiple organ failure)
11	25–30	TB	120	HL	Remission
12	15–20	TB	UK	HL	UK
13	10–15	ALCL	135	TB	Remission
14	65–70	TB	330	BALT lymphoma	Remission
15	65–70	*Mycobacterium avium* infection	420	BALT lymphoma	Remission
16	70–75	BALT lymphoma+TB	–	–	Remission
17	80–85	pneumonia	3	NHL + pulmonary aspergillosis	Died (respiratory failure)
18	65–70	NHL + CMV infection	–	–	Died (respiratory failure)

*NHL* non-Hodgkin’s lymphoma, *UK* unknown, *TB* tuberculosis, *HL* Hodgkin’s lymphoma, *DLBCL* diffuse large B-cell lymphoma, *ALCL* anaplastic large cell lymphoma, *BALT lymphoma* bronchus-associated lymphoid tissue lymphoma, *CMV* cytomegalovirus

The concurrence of lymphoma and TB is more common than concomitant fungal infection. The cellular immunodeficiency that usually accompanies lymphoma is believed to be a predisposing factor for opportunistic infection. But an aetiological role cannot be completely excluded. It has been reported that the risk of NHL is significantly increased in individuals with a history of TB, which is related to the DNA damage and apoptosis inhibition caused by *M. tuberculosis* [31–33]. It is not clear whether aspergillosis plays a similar role.

The concurrence of pulmonary lymphoma and opportunistic infection poses a management dilemma for physicians. A delayed diagnosis of either opportunistic infection or lymphoma usually occurs in this clinical scenario. The reasons can be summarized as follows: (1) Common symptoms and radiographic findings are shared by both disorders. When lymphoma and opportunistic infection exist simultaneously, it is difficult to judge whether the multifocal lesions and lymphadenopathy are lymphoma infiltrations or associated with infection. It makes the selection of the biopsy site challenging. (2) Tissue biopsy is the gold standard for the diagnosis and typing of lymphoma. While needle biopsy is inclusive, the majority of the tumour is constituted by reactive or inflammatory cells in varying compositions, especially HL. In HL, the neoplastic Hodgkin and Reed-Sternberg cells represent only a minority of the cellular infiltrate, with a frequency ranging from 0.1–10% [34]. Therefore, a single needle aspiration biopsy cannot ensure diagnostic yield.

The dilemma of lymphoma complicated with opportunistic infection is also reflected in treatment. Immediate application of chemotherapy may induce potential infection and aggravate the severity of the primary infection, especially for a particularly weak patient. Thus, it is necessary to identify whether evidence of infection exists in pathological specimens by specimen culture or special stains in addition to immunohistochemical staining. If anti-infectious treatments are given first, clinicians must notice that continuous antibiotic treatment for chronic infection may result in suppression of lymphoma and then create an illusion of clinical remission [27]. Therefore, when the treatment is less effective than expected or the clinical manifestations are not consistent with the infection, clinicians should look for other potential causes as soon as possible. If both diseases are treated at the same time, the risk of drug toxicity may be increased. So, choosing appropriate treatment timing is very important for these patients. Additional clinical data about the therapeutic plan for this condition should be collected to improve the prognosis.

In conclusion, simultaneous lymphoma and opportunistic infection in a primary presentation is a challenging condition. The diagnostic process should involve maintaining a high index of suspicion based upon an understanding of the clinical and imaging manifestations, of the therapeutic effect, and of the limitations of diagnostic methods. Different and various invasive diagnostic methods, including needle aspiration or excision biopsy of lymph nodes, CT-guided transthoracic needle aspiration, transbronchial biopsy and bone marrow puncture, should be performed to reach an early diagnosis.

## Data Availability

The datasets used and/or analysed during the current study are available from the corresponding author upon reasonable request.

## Abbreviations

ADA: Adenosine deaminase
ALCL: Anaplastic large cell lymphoma
BALF: Bronchoalveolar lavage fluid
BALT lymphoma: Bronchus-associated lymphoid tissue lymphoma
CMV: Cytomegalovirus
DLBCL: Diffuse large B-cell lymphoma
HL: Hodgkin’s lymphoma
HRCT: High-resolution computed tomography
IFI: Invasive fungal infection
LDH: Lactate dehydrogenase
NHL: Non-Hodgkin’s lymphoma
PAS: Periodic Acid-Schiff stain
PET-CT: Positron emission tomography/computed tomography
PPL: Primary pulmonary lymphoma
TB: Tuberculosis
TORCH: Toxoplasma, others, rubella virus, cytomegalovirus, herpes virus
UK: Unknown
